# Infection prevention and control practices in the Pediatrics and Child Health Department of Tikur Anbessa Specialized Hospital, Ethiopia

**DOI:** 10.3389/fpubh.2024.1329410

**Published:** 2024-01-19

**Authors:** Mengistu Yilma, Girma Taye, Muluwork Tefera, Berhan Tassew, Atalay Mulu Fentie, Workeabeba Abebe

**Affiliations:** ^1^Department of Epidemiology and Biostatistics, School of Public Health, College of Health Sciences, Addis Ababa University, Addis Ababa, Ethiopia; ^2^Department of Pediatrics and Child Health, School of Medicine, College of Health Sciences, Addis Ababa University, Addis Ababa, Ethiopia; ^3^Department of Health Systems and Health Policy, School of Public Health, College of Health Sciences, Addis Ababa University, Addis Ababa, Ethiopia; ^4^Department of Pharmacology and Clinical Pharmacy, School of Pharmacy, College of Health Sciences, Addis Ababa University, Addis Ababa, Ethiopia

**Keywords:** infection, prevention, control, healthcare workers, practices, pediatrics, child

## Abstract

**Background:**

Infection prevention and control (IPC) is a set of practices that are designed to minimize the risk of healthcare-associated infections (HAIs) spreading among patients, healthcare workers, and visitors. Implementation of IPC is essential for reducing infection incidences, preventing antibiotic use, and minimizing antimicrobial resistance (AMR). The aim of the study was to assess IPC practices and associated factors in Pediatrics and Child Health at Tikur Anbessa Specialized Hospital.

**Methods:**

In this study, we used a cross-sectional study design with a simple random sampling method. We determined the sample size using a single population proportion formula with the assumption of a 55% good IPC practice, a 5% accepted margin of error, and a 15% non-response rate and adjusted with the correction formula. The final sample size was 284 healthcare workers. The binary logistic regression model was used for analysis. The World Health Organization (WHO) Infection Prevention and Control Assessment Framework (IPCAF) tool was used to assess IPC core components.

**Result:**

A total of 272 healthcare workers participated in the study, with a response rate of 96%. Of the total participants, 65.8% were female and 75.7% were nurses. The overall composite score showed that the prevalence of good IPC practices among healthcare workers was 50.4% (95% CI: 44.3–56.5). The final model revealed that nursing professionals and healthcare workers who received IPC training had AORs of 2.84 (95% CI: 1.34–6.05) and 2.48 (95% CI: 1.36–4.52), respectively. The final average total IPCAF score for the IPC level was 247.5 out of 800 points.

**Conclusion:**

The prevalence of good IPC practice was suboptimal. The study participants, who were nursing professionals and healthcare workers who received IPC training, showed a statistically significant association with the IPC practice level. The facility-level IPCAF result showed a “Basic” level of practice based on the WHO categorization. These evidences can inform healthcare workers and decision-makers to identify areas for improvement in IPC practice at all levels. Training of healthcare workers and effective implementation of the eight IPC core components should be strengthened to improve suboptimal practices.

## Introduction

Infection prevention and control (IPC) is a set of practices that are designed to minimize the risk of healthcare-associated infections (HAIs) spreading among patients, healthcare workers, and visitors. Implementation of IPC is essential for reducing infection incidences, preventing antibiotic use, and minimizing antimicrobial resistance (AMR) (1, 2). An effective IPC program is critical for providing high-quality healthcare to patients and creating a safe working environment for those who work in health care settings (3).

A previously conducted study indicated that HAIs were reduced by 32% with the implementation of an effective IPC program for 5 years (4). Another study indicated that implementing effective IPC measures can reduce HAIs by up to 70% (5). HAIs are among the major public health issues that cause significant morbidity and mortality, lessen quality of life, and place a significant financial burden on patients and the healthcare system (5). Promoting simple IPC measures like good hand hygiene practices can reduce the AMR burden by up to 40% (6). Improving IPC can also reduce the number of HAIs that are causing prolonged hospital stays and massive financial losses (6).

Healthcare workers play a pivotal role in IPC by maintaining a safe and healthy environment for patients, themselves, and visitors. Adherence by healthcare providers to the basic IPC practices is critical for a successful IPC response. Individual and system-level IPC interventions must be comprehensive and multifaceted (7–10). The WHO recommends that all healthcare workers should implement IPC practices consistently when caring for all patients at all times and in all settings (11). However, healthcare facilities in low-income countries lack an effective IPC program due to inadequate water supply, sanitation, and environmental cleaning; insufficient equipment and supplies; understaffing and overcrowding; a lack of knowledge of all IPC measures; and the absence of IPC guidelines, policies, and programs (12, 13).

A systematic review and meta-analysis on the prevalence of standard precautions among healthcare workers in Low and Middle Income Countries (LMIC), in which 46 articles were included, showed a suboptimal practice with a pooled prevalence of 53.1% (14). From these 46 articles, a large number of studies assessed hand hygiene practice (41.3%), and the pooled prevalence hand hygiene was 59%, which wassuboptimal when compared with the WHO recommendation. The pooled prevalence of standard precautions (SP) among nurse professionals was 52.24%. The lowest prevalence of SP practice was 6.3%, which was about post-exposure prophylaxis utilization in Nigeria (15) and the highest prevalence was 98.6% about hand hygiene in Ethiopia (16).

Healthcare practice-associated occupational hazards like blood and body fluid exposure and sharp and needle stick injuries are the most common problems among healthcare workers. Some of the contributing factors to the occurrence of these hazards are: recapping of used needles; improper or no use of Personal Protective Equipment (PPE); poor handling and disposal of needles and sharps; not being vaccinated for Hepatitis B; and failing to follow safety instructions (17, 18). Needle stick injuries cause 39% of hepatitis C, 37% of hepatitis B, and 4.4% of HIV infections in healthcare workers (19). A meta-analysis and systematic review showed a high pooled incidence of needle stick injuries, with 43% occurring globally and 51% occurring in Africa (18). In Africa, the pooled lifetime and 12-months occupational exposures to body fluid were 65.7 and 48%, respectively (20). Occupational exposure to blood and body fluids is also common among Ethiopian healthcare workers, with a lifetime prevalence of 54.95% and a 12-month prevalence of 44.24% (21). Many LMICs pay less attention to the risk of transmission of *Mycobacterium tuberculosis* from patient to healthcare worker, and there are insufficient resources to prevent transmissions. According to the available evidence, the average prevalence of latent TB infections among HCWs was high (54% in LMICs) (19, 22).

In addition to healthcare worker-level IPC practices, facility-level practices are also crucial in implementing comprehensive and multifaceted IPC measures. The WHO recommended the implementation of the eight core components of IPC in all healthcare delivery facilities (23, 24). These are: the IPC program; IPC guidelines; IPC education; HAI surveillance; multimodal strategies; monitoring/auditing of IPC practices and feedback; workload staffing and bed occupancy; and environments, materials, and equipment for IPC (24). These core components scored the IPC level out of 800 total points, of which 100 points account for each component. The level scores were determined as “inadequate” (0–200), “basic” (201–400), “intermediate” (401–600), and “advanced” (601–800). WHO forwarded 11 recommendations and 3 good practice statements based on systematic reviews and expert consensus for core components of the IPC program (24, 25).

There were previous studies that assessed IPC practices in healthcare facilities; most of them focused on individual-level practices and were specific to single IPC components like hand hygiene (14). We did not find studies conducted in Ethiopia using the WHO IPCAF tool to assess the IPC level in health facilities that showed risks related to the environment. However, the current study incorporated more IPC practices, such as blood-borne infections, surgical wounds, urinary catheter management, and the use of personal protective equipment. Furthermore, we assessed work-related professional hazards (sharp and needle stick injuries, blood and body fluid exposure), and we evaluated the facility level IPC practices by using the IPCAF tool. The study was conducted by focusing on the pediatrics and child health areas. We hypothesized that the overall IPC practice level among healthcare workers in the study area is not different from the pooled prevalence (53.1%) in LMICs. To this end, the aim of the study was to determine the level of IPC practice among healthcare workers and at the facility level. This study can be important for understanding the magnitude of the problem, informing evidence-based decision-making, and identifying research gaps in the field of the IPC program.

## Methods and materials

Tikur Anbessa Specialized Hospital (TASH) is a teaching hospital for both clinical and preclinical fields. It is on the third tier of the Ethiopian health care delivery system and serves a population of 3.5 to 5 million. TASH is also an institution that provides specialized clinical services that are not available in other public health facilities in the nation. Pediatrics and Child Health is one of the departments in the hospital. This department provides services for an average of around 1,000 inpatients and 4,000 outpatients per month.

A cross-sectional study design was used to assess the IPC practices of healthcare workers in the Pediatrics and Child Health Department of Tikur Anbessa Specialized Hospital, Ethiopia. The data collection period for both healthcare worker and facility level assessments was from February to July 2022. All physicians and nurses who worked at the Pediatric and Child Health Department were included in the study. The healthcare workers who were on leave or training during the study period were excluded from the study. We used the simple random sampling technique to select participants by using the lists obtained from the human resources office.

We determined the sample size using a single population proportion formula with the assumption of a 55% good IPC practice (2 6), a 5% accepted margin of error, and a 15% non-response rate and adjusted with the correction formula.

Two trained data collectors collected the data during the working hours. The data were collected using an electronic format designed with ODK data collection software. The questionnaire was prepared in English and Amharic (the local language) side by side and uploaded to the data collectors’ tablets. The data were collected with the English version in reference to the Amharic version.

The study was conducted after obtaining ethical approval from the College of Health Science Institutional Review Board at Addis Ababa University with protocol number of 013/21/Pedi. Data from HCWs were collected after obtaining written informed consent and a brief description of the objectives of the study.

The completeness, accuracy, and consistency of the online submitted data were checked at the submission dates. After the data collection was completed, it was exported to Excel and opened with SPSS version 23 software for data management and analysis.

The binary logistic regression model was used to fit the bivariable and multivariable analyzes. Bivariable analyzes were conducted for each variable to check the association with the crude odds ratio, and then multivariable analyzes were fitted for all variables together to adjust the associations. For the association between outcome variable and explanatory variable, a 95% CI and an alpha level of <0.05 were considered to declare a statistically significant association. We checked for multicollinearity as one of the assumptions of binary logistic regression. The model’s fitness was checked with the Hosmer and Lemeshow tests. The proportion of good IPC practices among healthcare workers found in this study was tested against our hypothesis by using a nonparametric binomial test.

In this study, we used various categories of questions to define the outcome variable. These categories of questions were: blood-borne IPC practices (7 questions); surgical wound care practices (6 questions); hand hygiene practices (11 questions); urinary catheter management practice (5 questions); and personal protective equipment use practice (17 questions). A total of 46 questions were asked to determine the IPC practice level.

For the healthcare workers’ data, composite scores were computed for each category of questions based on the responses of healthcare workers. These scores were constructed by counting the number of “yes” responses in each case, and then the distribution of the responses was checked for normality to determine the appropriate measure of central tendency. Based on the normality test, the shape of the histogram and the Shapiro–Wilk test showed that the distribution of responses for all categories was negatively skewed. As a result, scores below the median were considered poor practice, and scores above and equal to the median were considered good practice.

Concerning facility-level IPC practices, a standardized WHO IPCAF tool was used to collect the data. The consecutive 6 months data were collected from the IPC unit of the hospital to ensure the reliability of the measurement. We added the subtotals that account for 100 points from each component to get an overall score of 800. Then we took the average score of the 6 months data to determine the IPC practice performance level of the facility as “inadequate” (0–200), “basic” (201–400), “intermediate” (401–600), or ‘advanced’ (601–800) (24).

## Results

### Socio-demographic characteristics

A total of 272 healthcare workers participated in the study, with a response rate of 96%. From the total participants, 65.8% were female, 75.7% were nurses, and only 29.8% had received training in the last 2 years from the time of data collection (Table 1).

**Table 1:** Healthcare worker’s socio-demographic characteristics in Pediatrics and Child Health Department of Tikur Anbessa Specialized Hospital, Addis Ababa, Ethiopia, 2022.

Characteristics		Frequency	Percent
Sex	Male	93	34.2
	Female	179	65.8
Age category	<=30 yrs	124	45.6
	>30 yrs	148	54.4
Profession	Nurse	206	75.7
	Physician	66	24.3
Marital status	Married	164	60.3
	Single	108	39.7
Years of experience	<= 5 yrs	117	43.0
	>5 yrs	155	57.0
IPC training last 2 yrs	Yes	81	29.8
	No	191	70.2
IPC guideline ever use	Always	32	11.8
	Rarely	172	63.2
	Not at all	68	25.0

### The healthcare workers’ IPC practices by components of practices

The overall composite score showed that the prevalence of good IPC practices among healthcare workers was 50.4% (95% CI: 44.3–56.5). The highest good score was recorded in urinary catheter IPC practice with 84.2% (95% CI: 79.3–88.3), and the lowest was in hand hygiene practice with 51.1% (95% CI, 45.0–57.2) (Table 2).

**Table 2:** IPC practices among healthcare workers by components of practices in Pediatrics and Child Health Department of Tikur Anbessa Specialized Hospital, Addis Ababa, Ethiopia, 2022.

Practice component		Frequency (%)	95% CI
Blood borne diseases IPC practice	Yes	62.5	56.5–68.3
	No	37.5	31.7–43.5
Surgical wound IPC practice	Yes	78.3	72.9–83.1
	No	21.7	16.9–27.1
Hand hygiene practice	Yes	51.1	45.0–57.2
	No	48.9	42.8–55.0
Urinary catheter IPC practice	Yes	84.2	79.3–88.3
	No	15.8	11.7–20.7
Use of personal protective equipment practice	Yes	51.1	45.0–57.2
	No	48.9	42.8–55.0
Overall composite score of IPC practices	Yes	50.4	44.3–56.5
	No	49.6	43.5–55.7

### Work related professional hazards among healthcare workers

From the total study participants, 115 (42.3%) were exposed to blood/body fluid and/or sharps/needle stick injuries in the last year. From the 48 study participants who were exposed to sharp/needle stick injuries in the last year, 15 (31.3%) were affected by sharps or needles visibly contaminated with blood. From those participants who were exposed to blood/body fluid and/or sharps/needles, only 8.7 and 6.1% received HIV and HepB post-exposure prophylaxis, respectively (Table 3).

**Table 3:** Work related professional hazards among healthcare workers in Pediatrics and Child Health Department of Tikur Anbessa Specialized Hospital, Addis Ababa, Ethiopia, 2022.

Work related hazards		Frequency (%)	95% CI
Blood/body fluid exposure in the 1 year	Yes	94 (34.6)	28.9–40.5
	No	178 (65.4)	59.5–71.1
Blood/body fluid exposure in the 1 month	Yes	37 (13.6)	9.8–18.3
	No	235 (86.4)	81.7–90.2
Sharp or needle stick injury in the last 1 year	Yes	41 (15.1)	11.0–19.9
	No	231 (84.9)	80.1–89
Sharp or needle stick injury in the last 1 month	Yes	17 (6.3)	3.7–9.8
	No	255 (93.7)	90.2–96.3
Sharp or needle visibly contaminated with blood	Yes	15 (31.3)	18.7–46.3
	No	33 (68.7)	53.7–81.3
Blood/body fluid, sharp, and needle exposure reported for supervisor	Yes	43 (37.4)	28.5–46.9
	No	72 (63.6)	53.1–71.5
Received HIV prophylaxis after exposures	Yes	10 (8.7)	4.2–15.4
	No	105 (91.3)	84.6–95.8
Received HepB prophylaxis after exposures	Yes	7 (6.1)	2.5–12.1
	No	108 (93.9)	87.9–97.5

### Factors associated with healthcare workers’ IPC practices

A binary logistic regression model with bivariable analysis showed a crude association between the dependent and the independent variables; as a result, five variables, namely sex, age category, profession, IPC training, and use of IPC guidelines, showed statistically significant associations. In a multivariable analysis, all variables were fitted to reveal the adjusted association by controlling confounding. The final model revealed that being a nurse and receiving IPC training in the last 2 years from the time of data collection had AORs of 2.84 (95% CI: 1.34–6.05) and 2.48 (95% CI: 1.36–4.52), respectively (Table 4).

**Table 4:** Healthcare workers’ IPC Practice and associated factors in Pediatrics and Child Health Department of Tikur Anbessa Specialized Hospital, Addis Ababa, Ethiopia, 2022.

Characteristics		IPC practice		Association	
		Good	Poor	Crude OR (95% CI)	Adjusted OR (95% CI)
Sex	Male	36	57	0.49 (0.29–0.81)*	0.81 (0.44–1.48)
	Female	101	78	1	1
Age category	<=30 yrs	50	74	0.47 (0.29–0.77)*	1.52 (0.78–2.96)
	>30 yrs	87	61	1	1
Profession	Nurse	118	88	3.32 (1.82–6.05)*	2.84 (1.34–6.05)**
	Physician	19	47	1	1
Marital status	Married	90	74	1.58 (0.97–2.58)	0.99 (0.55–1.79)
	Single	47	61	1	1
Years of experience	<= 5 yrs	51	66	0.62 (0.38–1.00)	1.52 (0.78–2.96)
	>5 yrs	85	70	1	1
IPC training in the last 2 years	Yes	57	24	3.30 (1.89–5.75)*	2.48 (1.36–4.52)**
	No	80	111	1	1
IPC guideline ever use	Always	17	15	1.83 (0.78–4.28)	1.39 (0.54–3.53)
	Rarely	94	78	1.95 (1.10–3.46)*	1.63 (0.87–3.03)
	Not at all	26	42	1	1

*significant for crude OR, **significant for adjusted OR.

### Health facility level IPC practices

We used aWHO IPCAF instrument to assess the eight core components of IPC practices at the facility level. We collected the consecutive 6 months’ data to assure measurement reliability. However, the data showed no variation in the performance of facility-level IPC practices within the 6 months. The final average total score was 247.5 out of 800 points, which was “basic” level performance as per the WHO categorization. The score of each individual component out of 100 points was as follows: IPC program (62.5), multimodal strategy (0), IPC guideline (7.5), IPC education and training (30), HAI surveillance (55), monitoring and audits of IPC practices and feedback (32.5), workload, staffing, and bed occupancy (15), and the built environment, materials, and equipment for IPC at the facility level (45).

## Discussion

Studying the current level of IPC practices and understanding their magnitude among healthcare workers at the healthcare facility level enables us to identify areas for improvement and plan targeted interventions and educational programs (26). According to our hypothesis, the prevalence of good IPC practices among healthcare workers (50.4%) in this study was not statistically different from the pooled prevalence of IPC practices (53.1%) in LMICs, with a value of p of 0.143. When compared to the WHO recommendation of high compliance rates with IPC practices, this finding revealed suboptimal good IPC practices among healthcare workers, although compliance can vary based on settings, types of health facilities, specific procedures, and local circumstances (11). Better IPC practices were expected in the current study setting since the hospital was specialized and could conduct high-level medical and surgical procedures.

In this study, the proportion of good IPC practice among healthcare workers was around half of the total study participants. This finding was slightly similar to other studies conducted in Ethiopia: a meta-analysis of 10 articles (52.2%) (27), a study in two teaching hospitals (55%) (28), a study in hospitals and health centers (54.2%) (29). Other studies conducted in Vietnam and Nigeria also showed a proportion of 48.1 and 51.1% of good IPC practices, respectively (30, 31).

This finding was also discordant with other study findings, both in increasing and decreasing directions. It was higher than studies conducted in hospitals in Hadiya Zone and hospitals in Gamo Gofa Zone of Ethiopia (39.8%) (32). The good IPC prevalence was higher than studies conducted in hospitals in Iran (19.5%) (8), and Cameron (19%) (33). The prevalence of good IPC practice in this study was lower than the study conducted in Gondar University referral hospital (34), and a hospital in Northwest Ethiopia (63.2%) (35). The possible reasons for this discrepancy could be the difference in components of IPC practices assessed, the number and types of healthcare workers who participated, the level of the health facility included in the studies, the IPC infrastructure, and the resources used by the health facilities, which could affect the practice level.

The assessment of occupational hazards in this study showed that a significant proportion of study participants (42.3%) were exposed to blood/body fluid (34.6%) and/or sharps/needle stick injuries (15.3%) in the last year and/or month from the time of data collection. From those participants who were exposed to blood/body fluid, and/or shards/needle stick injuries, only 8.7 and 6.1% received HIV and HepB post-exposure prophylaxis, respectively. The exposure levels in this study were lower than those in the study conducted in two teaching hospitals in the Amhara Region of Ethiopia, which were 56.7 and 36.3% of blood/body fluid and sharp/needle stick injuries in the last year from the time of data collection (28). The blood and body fluid exposures were also lower than the 12-month exposure levels (44.24%) and (48%) of the pooled prevalence from the systematic review and meta-analysis conducted in Ethiopia and Africa, respectively (20, 21). The blood/body fluid exposure level in this study was higher than the studies conducted in hospitals and health centers in Addis Ababa (16.5%) and Amhra Region (20.2%) (36, 37). The reasons for these discrepancies could be differences in the level of knowledge about occupational exposures and differences in adherence to standard precautions. The other possible reason could be under- or over-reporting of cases in different contexts. Understanding blood, body fluid, and sharp or needle stick exposure levels could be essential for healthcare workers to ensure safety and prevent the spread of infections. It is also important to consider universal precautions, post-exposure prophylaxis, training, and education in the context of the exposures.

The result of this study showed that the odds of good IPC practice among nurses was 2.84 times higher than among physicians. This indicates nurses had a higher likelihood of good IPC practice when compared with physicians. The finding of this study was supported by research conducted in Ethiopia (28). The reasons for the likelihood of more good IPC practice among nurses could be the feeling of vulnerability to infections, which makes them more concerned, and variation in receiving IPC-related training. The odds of good IPC practice was 2.48 times higher among healthcare workers who received IPC training in the last 2 years from the time of the data collection than their counterparts. This finding was consistent with studies conducted in the Wolaitta Sodo teaching referral hospital (38), Southeast Ethiopia (39), Northeast Ethiopia (40). This indicates that training is of utmost importance for healthcare workers to equip them with knowledge about proper use of personal protective equipment, hand hygiene, and other infection control measures. The training encourages continuous learning and improvement that can help healthcare workers stay updated on the latest evidence-based practices, strategies, and technologies related to IPC.

The facility-level IPC practices assessed in this study revealed that the practice level in Tikur Anbessa specialized hospital was “Basic” IPC level, with an average overall IPCAF score of 247.5 out of 800 points. This assessment can provide fundamental insight about the level of implementation of the eight IPC core components in the hospital. This facility-level practice score could also have a direct effect on the pediatrics and child health department of the hospital. The “Basic” IPC level scored in this assessment was consistent with the study conducted in Bangladesh within 11 tertiary-care hospitals that showed a “Basic” IPC level with a 355 overall score out of 800 points (41) and the global survey conducted by WHO that showed a “Basic” IPC level in low income countries (42). According to the WHO categorization, “Basic” IPC level with an IPCAF score of 201–400 means some of the IPC core components are in place but not fully implemented, and more improvement is required (24). Although studies revealed that effective implementation of IPC practices can reduce the incidence of HAIs by up to 70% in healthcare facilities (5), the assessment in the current facility showed that only “Basic” IPC level implementation was met. As we conducted the assessment for 6 months by collecting data every month, no improvement was recorded across these time points. The lowest score was recorded in core component 5 (Multimodal strategy), which was not applied at all, while the highest score was recorded in core component 1 (IPC program). Since TASH is a specialized referral and teaching hospital that serves a large number of critical cases and performs high-level medical and surgical procedures, it should achieve at least “Intermediate” IPC-level practice to ensure the safety of patients and healthcare workers as well as mitigating the spread of infections.

### Strengths and limitations

This study was comprehensive and assessed both healthcare worker and facility-level IPC practices as well as occupational hazards among health workers. We conducted six consecutive months of data collection using the IPCAF tool for the facility-level IPC practice assessment to ensure the reliability of the measurements. The study was hypothesis-based and tested accordingly.

The limitations of this study include that it was conducted in a single hospital, which may not be generalizable to other hospitals; the healthcare workers’ IPC practice level was assessed with self-reported information, which may not reflect the actual practice level; and the cross-sectional nature of the study may not reflect the cause-and-effect relationship.

## Conclusion

The prevalence of good IPC practices was suboptimal when compared with the WHO recommendations and the findings of some other studies conducted previously. A significant proportion of healthcare workers were exposed to blood/body fluid, and/or sharps/needle stick injuries in the past year from the time of data collection, and a small proportion of them took post-exposure prophylaxis for HIV and/or HepB. The study participants’ profession and training on IPC in the last 2 years from the time of data collection revealed a statistically significant association with the IPC practice level of healthcare workers. The facility’s IPC practice level was “Basic” which indicated some of the IPC core components are in place but not effectively implemented, and more improvement is required. These low-level IPC practices of the healthcare workers and the facility should be enhanced by providing IPC training and effective implementation of the eight IPC core components as per the WHO recommendations. The healthcare practices associated occupational hazards among healthcare workers also need special attention to ensure their safety.

The authors will communicate the findings of this study to the hospital and the pediatrics department to initiate and promote interventions based on the results. This can serve as a baseline to adapt possible interventions like training and mentorship to improve IPC practices at the healthcare worker and facility level.

## Data availability statement

The raw data supporting the conclusions of this article will be made available by the authors, without undue reservation.

## Ethics statement

The studies involving humans were approved by Addis Ababa University, College of Health Sciences Institutional Review Board. The studies were conducted in accordance with the local legislation and institutional requirements. The participants provided their written informed consent to participate in this study. Written informed consent was obtained from the individual(s) for the publication of any potentially identifiable images or data included in this article.

## Author contributions

MY: Conceptualization, Data curation, Formal analysis, Investigation, Methodology, Writing – original draft, Writing – review & editing. GT: Supervision, Validation, Writing – review & editing. WA: Conceptualization, Supervision, Validation, Writing – review & editing. MT: Writing – review & editing. BT: Writing – review & editing. AF: Writing – review & editing.
