# Supplementary material for: Infection prevention and control practices in the Pediatrics and Child Health Department of Tikur Anbessa Specialized Hospital, Ethiopia
Source: Front Public Health. 2024 Jan 19;12:1329410. doi: 10.3389/fpubh.2024.1329410 (PMC10834730; doi:10.3389/fpubh.2024.1329410)
Supplement: Supplementary file 1 [file Table_1.DOCX]

## Health workers IPC practice assessment tool

| **Assess**  **the existing level of infection prevention and control** **implementation** | |
| --- | --- |
| Date of interview | |
| Interviewer name | |
| Interviewee code [____\| | |
| Healthcare worker’s socio-demographic characteristics | |
| 1. Age of the respondent (years) |  |
| 1. Gender | Female |
|  | Male |
| 1. Marital status | Single |
|  | Married |
|  | Widowed |
|  | Divorced |
| 1. Profession | Physician  Nurse  Health officer  Laboratory  other |
| 1. Year of experience |  |
| ***Healthcare workers’ IPC practice*** | |
| 1. Did you take any IPC training in the past 2 years? | Yes  No |
| 1. Did you use/refer the hospital IPC Guideline ever? | Always  Rarely  Not at all |
| 1. Did you face any blood/body fluid exposure in the past one year? | Yes  No |
| 1. Did you face any Blood /body fluids in the past one month? | Yes  No |
| 1. Did you face any sharp or needle stick injury in the past one year? | Yes  No |
| 1. Did you face any sharp or needle stick injury in the past one month? | Yes  No 🡪15 |
| 1. Was the sharp or needle visibly contaminated with blood prior to exposure? | Yes  No |
| 1. Did you report the exposure for your supervisor promptly after exposure? | Yes  No |
| 1. Did you receive HIV/AIDS prophylaxis after exposure? | Yes  No |
| 1. Did you receive Hepatitis prophylaxis after exposure? | Yes  No |
| 1. Are you instructed to report signs and symptoms of infectious condition for the supervisor? | Yes  No |
| 1. Blood borne diseases practice    1. Are you avoiding direct contact with blood?    2. Are you avoiding recapping and bending of needles?    3. Do you always wear pair glove when caring patients?    4. Do you wear mask and protective eye-wear when splash of blood or body fluid is anticipated?    5. Do you wiped off any spillage of blood and body fluid with cloths/towels soaked in disinfectant solution?    6. Do you place blood-soiled gloves, dressing, cotton, cloths etc in plastic bags for disposal?    7. Do you dispose sharps and needles in safety boxes? | Yes No  Yes No  Yes No  Yes No  Yes No    Yes No  Yes No |
| 1. Surgical wound infection practice    1. Do you avoiding wounds with bare hands?    2. Do you apply antiseptic as necessary?    3. Do you prescribe proper antibiotic for wounds?    4. Do you rinse the wound with salt water as necessary?    5. Do you drain the pocket of pus (abscess), if present?    6. Do you apply VAC (vacuum assisted closure) dressing to facilitate healing? | Yes No  Yes No  Yes No  Yes No  Yes No  Yes No |
| 1. Urinary catheter practice    1. Do you keep the drainage bag below the level of bladder?    2. Do you instruct the patients to keep the drainage bag off floor at all times?    3. Do you keep the catheter secured to the patients’ thigh to avoid movement of it?    4. Do you instruct patients not to lie on the catheter or block the line?    5. Do you instruct patients to keep the catheter clean? | Yes No  Yes No  Yes No  Yes No  Yes No |
| 1. Hand hygiene practice    1. Do you wash your hands before and after examining (coming in direct contact with) a client/patient?    2. Do you wash your hands immediately after touching contaminated instruments or articles?    3. Do you wash your hands immediately after exposure/bare skin contact to mucous membranes, blood, body fluids secretions or excretions?    4. Do you wash your hands before putting on gloves and after removing them?    5. Do you wash your hands immediately on arrival and departure from work (the health facility    6. Do you wash hands whenever the hands become visibly soiled?    7. Do you Cover superficial cuts or open skin lesions with a waterproof dressing, even if gloves are worn over the affected area/s?    8. Do you refrain from direct patient contact if you have an exudative lesion or weeping dermatitis on the lower arms, hands or face that cannot effectively be dressed to prevent transmission, until the condition is resolved?    9. Do you use an aqueous based hand moisturizer regularly to maintain skin health; compatible with the hand hygiene products used?    10. Do you Keep fingernails short and clean?    11. Do you refrain from wearing nail polish, nail jewellery, artificial nails, and jewellery on the hands or arms? | Yes No  Yes No  Yes No    Yes No  Yes No  Yes No  Yes No  Yes No  Yes No  Yes No    Yes No |

| 1. Use of PPE    1. Do you wear properly fitting disposable gloves for all patients?    2. Do you use a new pair of gloves for each patient?    3. Do you replace gloves as soon as possible if they become soiled or damaged, do not wash gloves as this may damage glove integrity?    4. Do you wear examination gloves during any medical procedures?    5. Do you wear a fluid-resistant mask that meets the minimum standard?    6. Do you fit and wear your mask in accordance with the manufacturer’s instructions, ensuring an adequate seal around both the nose and mouth. Avoid touching the front of the mask during patient treatment?    7. Do you change your mask between patients and when damp or visibly contaminated during treatment. Remove by touching the strings and loops only, and discard immediately after use?    8. Do you remove gloves, masks and protective eyewear before moving from a contaminated zone to a clean zone in your practice setting?    9. Do you use sequencing that minimises the spread of contamination when donning and removing personal protective equipment?    10. Do you wear protective eyewear that is fit for purpose, and is optically clear and distortion free?    11. Do you wear a mask with the face shield to provide protection against inhalation of micro-organisms?    12. Do you wear outer protective clothing (for example, gowns, tunics) over your street clothing or uniform. Outer protective clothing is to be made from material that does not permit blood or other potentially infectious materials to reach clothes or skin underneath?    13. Do you change outer protective clothing: as soon as possible when visibly soiled or wet, when exposed to contaminated aerosols for prolonged periods of time, and at least daily?    14. Do you remove outer protective clothing before leaving the treatment area for: a break involving eating and/or drinking, a toilet break, and before leaving the practice premises?    15. Do you launder reusable outer protective clothing properly?    16. Do you place disposable outer protective clothing in the controlled waste after use, unless it is contaminated with blood to the extent that it qualifies as hazardous waste?    17. Do you wear enclosed footwear that will protect your feet against injury from sharp objects and contamination when necessary? | Yes No  Yes No  Yes No    Yes No  Yes No  Yes No    Yes No  Yes No  Yes No  Yes No  Yes No  Yes No    Yes No  Yes No  Yes No    Yes No  Yes No |
| --- | --- |

## Infection Prevention and Control Assessment Tool at facility level

| **This questionnaire should be filled for the Department of Pediatrics and Child Health in TASH.** | | |
| --- | --- | --- |
| **Survey information** | | |
| Date of interview (dd/mm/yyyy) [___\|___]-[___\|___]-[ 2\| 0 \| 2\| 1] | | |
| Interviewers name [_____________________________________________] interviewers code [____\| | | |
| **Core component 1: Infection Prevention and Control (IPC) programme** | | |
| **Questions** | **Answers** | **Score** |
| **Do you have an IPC Program?**  CIRCLE ONE ANSWER | **0 No** | **0** |
|  | **1 Yes, without clearly defined objectives** | **5** |
|  | **2 Yes, with clearly defined objectives and annual activity plan** |  |
| - - - 1. Is the IPC program supported by an IPC team comprising of IPC professionals?   CIRCLE ONE ANSWER | **0 No** | **0** |
|  | **1 Not a team, only an IPC focal person** | **5** |
|  | **2 Yes** | **10** |
| - - - 1. Does the IPC team have at least one full-time IPC professional or equivalent (nurse or doctor working 100% in IPC) available?   CIRCLE ONE ANSWER | **0 No IPC professional available** | **0** |
|  | **1 No, only part-time professional available** | **2.5** |
|  | **2 Yes, One per >250 beds** | **5** |
|  | **3 Yes, One per<=250 beds** | **10** |
| 4. Does the IPC team or focal person have dedicated time for IPC activities? | **0 No** | **0** |
|  | **1 Yes** | **10** |
| 5. Does the IPC team include both doctors and nurses? | **0 No** | **0** |
|  | **1 Yes** | **10** |
| 6. Do you have an IPC committee5 actively supporting the IPC team? | **0 No** | **0** |
|  | **1 Yes** | **10** |
| **7. Are any of the following professional groups represented/included in the IPC committee?** | | |
| Senior facility leadership (for example, administrative director, chief executive officer [CEO], medical director) | 0 No | 0 |
|  | 1 Yes | 10 |
| Senior clinical staff (for example, physician, nurse) | 0 No | 0 |
|  | 1 Yes | 10 |
| Facility management (for example, biosafety, waste, and those tasked  with addressing water, sanitation, and hygiene [WASH]) | 0 No | 0 |
|  | 1 Yes | 10 |
| **8. Do you have clearly defined IPC objectives (that is, in specific critical areas)?**  CIRCLE ONE ANSWER | 0 No | 0 |
|  | 1 Yes, IPC objectives only | 2.5 |
|  | 2 Yes, IPC objectives and measurable outcome indicators (that is, adequate measures for improvement) | 5 |
|  | 3 Yes, IPC objectives, measurable outcome indicators and set future targets | 10 |
| **9. Does the senior facility leadership show clear commitment and support for the IPC programme:** | |  |
| By an allocated budget specifcally for the IPC programme (that is, covering IPC activities, including salaries)? | 0 No | 0 |
|  | 1 Yes | 5 |
| By demonstrable support for IPC objectives and indicators within the facility (for example, at executive level meetings, executive rounds, participation in morbidity and mortality meetings)? | 0 No | 0 |
|  | 1 Yes | 5 |
| **10. Does your facility have microbiological laboratory support (either present on or off site) for routine day-to-day use?**  CIRCLE ONE ANSWER | 0 No | 0 |
|  | 1 Yes, but not delivering results reliably (timely and of sufficient quality) | 5 |
|  | 2 Yes, and delivering results reliably (timely and of sufficient quality) | 10 |
| **Subtotal Score /100** | | |

| **Core component 2: Infection Prevention and Control (IPC) guidelines** | | |
| --- | --- | --- |
| Question | Answer | score |
| 1 Does your facility have the expertise (in IPC and/or infectious diseases) for developing or adapting guidelines? | 0 No | 0 |
|  | 1 Yes | 7.5 |
| **2 Does your facility have guidelines available for:** | | |
| Standard precautions? | 0 No | 0 |
|  | 1 Yes | 2.5 |
| Hand hygiene? | 0 No | 0 |
|  | 1 Yes | 2.5 |
| Transmission-based precautions? | 0 No | 0 |
|  | 1 Yes | 2.5 |
| Outbreak management and preparedness? | 0 No | 0 |
|  | 1 Yes | 2.5 |
| Prevention of surgical site infection? | 0 No | 0 |
|  | 1 Yes | 2.5 |
| Prevention of vascular catheter-associated bloodstream infections? | 0 No | 0 |
|  | 1 Yes | 2.5 |
| Prevention of hospital-acquired pneumonia ([HAP]; all types of HAP, including (but not exclusively) ventilator-associated pneumonia)? | 0 No | 0 |
|  | 1 Yes | 2.5 |
| Prevention of catheter-associated urinary tract infections? | 0 No | 0 |
|  | 1 Yes | 2.5 |
| Prevention of transmission of multidrug-resistant (MDR) pathogens? | 0 No | 0 |
|  | 1 Yes | 2.5 |
| Disinfection and sterilization? | 0 No | 0 |
|  | 1 Yes | 2.5 |
| Health care worker protection and safety? | 0 No | 0 |
|  | 1 Yes | 2.5 |
| Injection safety? | 0 No | 0 |
|  | 1 Yes | 2.5 |
| Waste management? | 0 No | 0 |
|  | 1 Yes | 2.5 |
| Antibiotic stewardship? | 0 No | 0 |
|  | 1 Yes | 2.5 |
| **3 Are the guidelines in your facility consistent with national/international guidelines (if they exist)?** | 0 No | 0 |
|  | 1 Yes | 10 |
| **4. Is implementation of the guidelines adapted10 according to the local needs and resources while maintaining key IPC standards?** | 0 No | 0 |
|  | 1 Yes | 10 |
| **5. Are frontline health care workers involved in both planning and executing the implementation of IPC guidelines in addition to IPC personnel?** | 0 No | 0 |
|  | 1 Yes | 10 |
| **6. Are relevant stakeholders (for example, lead doctors and nurses, hospital managers, quality management) involved in the development and adaptation of the IPC guidelines in addition to IPC personnel?** | 0 No | 0 |
|  | 1 Yes | 10 |
| **7. Do health care workers receive specific training related to new or updated IPC guidelines introduced in the facility?** | 0 No | 0 |
|  | 1 Yes | 10 |
| **8. Do you regularly monitor the implementation of at least some of the IPC guidelines in your facility?** | 0 No | 0 |
|  | 1 Yes | 10 |
| **Subtotal score** **/100** | | |

| **Core component 3: Infection Prevention and Control (IPC) education and training** | | |
| --- | --- | --- |
| **Questions** | **Answers** | **Score** |
| **1. Are there personnel with the IPC expertise (in IPC and/or infectious diseases) to lead IPC training?** | 0 No | 0 |
|  | 1 Yes | 10 |
| **2. Are there additional non-IPC personnel with adequate skills to serve as trainers and mentors (for example, link nurses or doctors, champions)?**  **CIRCLE ONE ANSWER** | 0 No | 0 |
|  | 1 Yes | 10 |
| **3. How frequently do health care workers receive training regarding IPC in your facility?**  **CIRCLE ONE ANSWER** | 0 Never or rarely | 0 |
|  | 1 New employee orientation only for health care workers | 5 |
|  | 2 New employee orientation and regular (at least annually) IPC training for health care workers offered but not mandatory | 10 |
|  | 3 New employee orientation and regular (at least annually) mandatory IPC training for all health care workers | 15 |
| **4. How frequently do cleaners and other personnel directly involved in patient care receive training regarding IPC in your facility**  CIRCLE ONE ANSWER | 0 Never or rarely | 0 |
|  | 1 New employee orientation only for other personnel | 5 |
|  | 2 New employee orientation and regular (at least annually) training for other personnel offered but not mandatory | 10 |
|  | 3 New employee orientation and regular (at least annually) mandatory IPC training for other personnel | 15 |
| **5. Does administrative and managerial staff receive general training regarding IPC in your facility?**  **CIRCLE ONE ANSWER** | 0 No | 0 |
|  | 1 Yes | 5 |
| **6 How are health care workers and other personnel trained**  CIRCLE ONE ANSWER | 0 No training available | 0 |
|  | 1 Using written information and/or oral instruction and/or e-learning only | 5 |
|  | 2 Includes additional interactive training sessions (for example, simulation and/or bedside training) | 10 |
| **7. Are there periodic evaluations of the effectiveness of training programmes (for example, hand hygiene audits, other checks on knowledge)?**  CIRCLE ONE ANSWER | 0 No | 0 |
|  | 1 Yes, but not regularly | 5 |
|  | 2 Yes, regularly (at least annually) | 10 |
| **8. Is IPC training integrated in the clinical practice and training of other specialties (for example, training of surgeons involves aspects of IPC)?**  CIRCLE ONE ANSWER | 0 No | 0 |
|  | 1 Yes, in some disciplines | 5 |
|  | 2 Yes, in all disciplines | 10 |
| **9. Is there specifc IPC training for patients or family members to minimize the potential for health care-associated infections (for example, immunosuppressed patients, patients with invasive devices, patients with multidrug-resistant infections)?** | 0 No | 0 |
|  | 1 Yes | 5 |
| **10. Is ongoing development/education offered for IPC staff (for example, by regularly attending conferences, courses)?** | 0 No | 0 |
|  | 1 Yes | 10 |
| **Subtotal score /100** | | |

| **Core component 4: Health care-associated infection (HAI) surveillance** | | |
| --- | --- | --- |
| **Question** | **Answer** | **score** |
| **Organization of surveillance** | | |
| **1. Is surveillance a defined component of your IPC programme?** | 0 No | 0 |
|  | 1 Yes | 5 |
| **2. Do you have personnel responsible for surveillance activities?** | 0 No | 0 |
|  | 1 Yes | 5 |
| **3. Have the professionals responsible for surveillance activities been trained in basic epidemiology, surveillance and IPC (that is, capacity to oversee surveillance methods, data management and interpretation)?** | 0 No | 0 |
|  | 1 Yes | 5 |
| **4. Do you have informatics/IT support to conduct your surveillance (for example, equipment, mobile technologies, electronic health records)?** | 0 No | 0 |
|  | 1 Yes | 5 |
| **Priorities for surveillance - defined according to the scope of care** | | |
| **5. Do you go through a prioritization exercise to determine the HAIs to be targeted for surveillance according to the local context (that is, identifying infections that are major causes of morbidity and mortality in the facility)?** | 0 No | 0 |
|  | 1 Yes | 5 |
| **6. In your facility is surveillance conducted for:** | | |
| Surgical site infections? | 0 No | 0 |
|  | 1 Yes | 2.5 |
| Device-associated infections (for example, catheter-associated urinary tract infections, central line-associated bloodstream infections, peripheral-line associated bloodstream infections, ventilator-associated pneumonia)? | 0 No | 0 |
|  | 1 Yes | 2.5 |
| Clinically-defined infections (for example, definitions based only on clinical signs or symptoms in the absence of microbiological testing)? | 0 No | 0 |
|  | 1 Yes | 2.5 |
| Colonization or infections caused by multidrug-resistant13 pathogens according to your local epidemiological situation? | 0 No | 0 |
|  | 1 Yes | 2.5 |
| Local priority epidemic-prone infections (for example, norovirus, influenza, tuberculosis [TB], severe acute respiratory syndrome [SARS], Ebola, Lassa fever)? | 0 No | 0 |
|  | 1 Yes | 2.5 |
| Infections in vulnerable populations (for example, neonates, intensive care unit, immunocompromised, burn patients)? | 0 No | 0 |
|  | 1 Yes | 2.5 |
| Infections that may affect health care workers in clinical, laboratory, or other settings (for example, hepatitis B or C, human immunodeficiency virus [HIV], influenza)? | 0 No | 0 |
|  | 1 Yes | 2.5 |
| **7. Do you regularly evaluate if your surveillance is in line with the current needs and priorities of your facility?** | 0 No | 0 |
|  | 1 Yes | 5 |
| **Methods of surveillance** | | |
| **8. Do you use reliable surveillance case definitions (defined numerator and denominator according to international definitions?** | 0 No | 0 |
|  | 1 Yes | 5 |
| **9. Do you use standardized data collection methods (for example, active prospective surveillance) according to international surveillance protocols (for example, CDC NHSN/ECDC) or if adapted, through an evidence-based adaptation process and expert consultation?** | 0 No | 0 |
|  | 1 Yes | 5 |
| **10. Do you have processes in place to regularly review data quality (for example, assessment of case report forms, review of microbiology results, denominator determination, etc.)?** | 0 No | 0 |
|  | 1 Yes | 5 |
| **11. Do you have adequate microbiology and laboratory capacity to support surveillance?**  CIRCLE ONE ANSWER | 0 No | 0 |
|  | 1 Yes, can differentiate gram positive/negative strains but cannot identify pathogens | 2.5 |
|  | 2 Yes, can reliably identify pathogens (for example, isolate identification) in a timely manner | 5 |
|  | 3 Yes, can reliably identify pathogens and antimicrobial drug resistance patterns (that is, susceptibilities) in a timely manner | 10 |
| **Information analysis and dissemination/data use, linkage, and governance** | | |
| **12. Are surveillance data used to make tailored unit/facility-based plans for the improvement of IPC practices?** | 0 No | 0 |
|  | 1 Yes | 5 |
| **13. Do you analyze antimicrobial drug resistance on a regular basis (for example, quarterly/half-yearly/annually)?** | 0 No | 0 |
|  | 1 Yes | 5 |
| **14. Do you regularly (for example, quarterly/half-yearly/annually) feedback up-to-date surveillance information to:** | | |
| Frontline health care workers (doctors/nurses)? | 0 No | 0 |
|  | 1 Yes | 2.5 |
| Clinical leaders/heads of department | 0 No | 0 |
|  | 1 Yes | 2.5 |
| IPC committee | 0 No | 0 |
|  | 1 Yes | 2.5 |
| Non-clinical management/administration (chief executive officer/chief financial officer)? | 0 No | 0 |
|  | 1 Yes | 2.5 |
| **15. How do you feedback up-to-date surveillance information? (at least annually)** | 0 No feedback | 0 |
|  | 1 By written/oral information only | 2.5 |
|  | 2 By presentation and interactive problem-orientated solution finding | 7.5 |
| **Subtotal score /100** | | |

| **Core component 5: Multimodal strategies16 for implementation of infection prevention and control (IPC) interventions** | | |
| --- | --- | --- |
| **Question** | **Answer** | **score** |
| **1. Do you use multimodal strategies16 to implement IPC interventions?** | 0 No | 0 |
|  | 1 Yes | 15 |
| **2. Do your multimodal strategies include any or all of the following elements:**  CIRCLE ONE ANSWER (THE MOST ACCURATE) PER ELEMENT | **System change** | |
|  | 0 Element not included in multimodal strategies | 0 |
|  | 1 Interventions to ensure the necessary infrastructure and continuous availability of supplies are in place | 5 |
|  | 2 Interventions to ensure the necessary infrastructure and continuous availability of supplies are in place and addressing ergonomics17 and accessibility, such as the best placement of central venous catheter set and tray | 10 |
|  | **Education and training** | |
|  | 0 Element not included in multimodal strategies | 0 |
|  | 1 Written information and/or oral instruction and/or e-learning only | 5 |
|  | 2 Additional interactive training sessions (includes simulation and/or bedside training) | 10 |
|  | **Monitoring and feedback** | |
|  | 0 Element not included in multimodal strategies | 0 |
|  | 1 Monitoring compliance with process or outcome indicators (for example, audits of hand hygiene or catheter practices) | 5 |
|  | 2 Monitoring compliance and providing timely feedback of monitoring results to health care workers and key players | 10 |
|  | **Communications and reminders** | |
|  | 0 Element not included in multimodal strategies | 0 |
|  | 1 Reminders, posters, or other advocacy/awareness-raising tools to promote the intervention | 5 |
|  | 2 Additional methods/initiatives to improve team communication across units and disciplines (for example, by establishing regular case conferences and feedback rounds) | 10 |
|  | **Safety climate and culture change** | |
|  | 0 Element not included in multimodal strategies | 0 |
|  | 1 Managers/leaders show visible support and act as champions and role models, promoting an adaptive approach18 and strengthening a culture that supports IPC, patient safety and quality | 5 |
|  | 2 Additionally, teams and individuals are empowered so that they perceive ownership of the intervention (for example, by participatory feedback rounds) | 10 |
| **3. Is a multidisciplinary team used to implement IPC multimodal strategies?** | 0 No | 0 |
|  | 1 Yes | 15 |
| **4. Do you regularly link to colleagues from quality improvement and patient safety to develop and promote IPC multimodal strategies?** | 0 No | 0 |
|  | 1 Yes | 10 |
| **5. Do these strategies include bundles19 or checklists?** | 0 No | 0 |
|  | 1 Yes | 10 |
| **Subtotal score /100** | | |

| **Core component 6: Monitoring/audit of IPC practices and feedback** | | |
| --- | --- | --- |
| **Question** | Answer | Score |
| **1. Do you have trained personnel responsible for monitoring/audit of IPC practices and feedback?** | 0 No | 0 |
|  | 1 Yes | 10 |
| **2. Do you have a well-defined monitoring plan with clear goals, targets and activities (including tools to collect data in a systematic way)?** | 0 No | 0 |
|  | 1 Yes | 7.5 |
| **3. Which processes and indicators do you monitor in your facility?**  CIRCLE ALL THAT APPLY | 0 None | 0 |
|  | 1 Hand hygiene compliance (using the WHO hand hygiene observation tool20 or equivalent) | 5 |
|  | 2 Hand hygiene compliance (using the WHO hand hygiene observation tool20 or equivalent) | 5 |
|  | 3 Wound dressing change | 5 |
|  | 4 Transmission-based precautions and isolation to prevent the spread of multidrug resistant organisms (MDRO) | 5 |
|  | 5 Cleaning of the ward environment | 5 |
|  | 6 Disinfection and sterilization of medical equipment/instruments | 5 |
|  | 7 Consumption/usage of alcohol-based hand rub or soap | 5 |
|  | 8 Consumption/usage of antimicrobial agents | 5 |
|  | 9 Waste management | 5 |
| **4. How frequently is the WHO Hand Hygiene Self-Assessment Framework Survey 21 undertaken?**  **CIRCLE ONE ANSWER** | 0 Never | 0 |
|  | 1 Periodically, but no regular schedul | 2.5 |
|  | 2 At least annually | 5 |
| **5. Do you feedback auditing reports (for example, feedback on hand hygiene compliance data or other processes) on the state of the IPC activities/performance?**  CIRCLE ALL THAT APPLY | 0 No reporting | 0 |
|  | 1 Yes, within the IPC team | 2.5 |
|  | 2 Yes, to department leaders and managers in the areas being audited | 2.5 |
|  | 3 Yes, to frontline health care workers | 2.5 |
|  | 4 Yes, to the IPC committee or quality of care committees or equivalent | 2.5 |
|  | 5 Yes, to hospital management and senior administration | 2.5 |
| **6. Is the reporting of monitoring data undertaken regularly (at least annually)?** | 0 No | 0 |
|  | 1 Yes | 10 |
| **7. Are monitoring and feedback of IPC processes and indicators performed in a “blame-free” institutional culture aimed at improvement and behavioural change?** | 0 No | 0 |
|  | 1 Yes | 10 |
| **8. Do you assess safety cultural factors in your facility (for example, by using other surveys such as HSOPSC, SAQ, PSCHO, HSC22)** | 0 No | 0 |
|  | 1 Yes | 5 |
| **Subtotal score /100** | | |

| **Core component 7: Workload, staffing and bed occupancy** | | |
| --- | --- | --- |
| **Question** | Answer | Score |
| **Staffing** | | |
| **1. Are appropriate staffing levels assessed in your facility according to patient workload using national standards or a standard staffing needs assessment tool such as the WHO Workload indicators of staffing need24 method?** | 0 No | 0 |
|  | 1 Yes | 5 |
| **2. Is an agreed (that is, WHO or national) ratio of health care workers to patients25 maintained across your facility?**  CIRCLE ONE ANSWER | 0 No | 0 |
|  | 1 Yes, for staff in less than 50% of units | 5 |
|  | 2 Yes, for staff in more than 50% of units | 10 |
|  | 3 Yes, for all health care workers in the facility | 15 |
| **3. Is a system in place in your facility to act on the results of the staffing needs assessments when staffing levels are deemed to be too low?** | 0 No | 0 |
|  | 1 Yes | 10 |
| **Bed occupancy** | | |
| **4. Is the design of wards in your facility in accordance with international standards26 regarding bed capacity?**  CIRCLE ONE ANSWER | 0 No | 0 |
|  | 1 Yes, but only in certain departments | 5 |
|  | 2 Yes, for all departments (including emergency department and pediatrics) | 15 |
| **5. Is bed occupancy in your facility kept to one patient per bed?** | 0 No | 0 |
|  | 1 Yes, but only in certain departments | 5 |
|  | 2 Yes, for all units (including emergency departments and pediatrics) | 15 |
| **6. Are patients in your facility placed in beds standing in the corridor outside of the room (including beds in the emergency department)?**  CIRCLE ONE ANSWER | 0 No | 0 |
|  | 1 Yes, more frequently than twice a week | 5 |
|  | 2 Yes, less frequently than twice a week | 10 |
| **7. Is adequate spacing of > 1 meter between patient beds ensured in your facility?**  CIRCLE ONE ANSWER | 0 No | 0 |
|  | 1 Yes, but only in certain departments | 5 |
|  | 2 Yes, for all departments (including emergency department and pediatrics) | 15 |
| **8. Is a system in place in your facility to assess and respond when adequate bed capacity is exceeded?**  CIRCLE ONE ANSWER | 0 No | 0 |
|  | 1 Yes, this is the responsibility of the head of department | 5 |
|  | 2 Yes, this is the responsibility of the hospital administration/ management | 10 |
| **Subtotal score /100** | | |

| **Core component 8: Built environment, materials and equipment for IPC at the facility level** | | |
| --- | --- | --- |
| **Question** | Answer | Score |
| **Water** | | |
| **1. Are water services available at all times and of sufficient quantity for all uses (for example, hand washing, drinking, personal hygiene, medical activities, sterilization, decontamination, cleaning and laundry)?**  CIRCLE ONE ANSWER | 0 No, available on average < 5 days per week | 0 |
|  | 1 Yes, available on average ≥ 5 days per week or every day but not of sufficient quantity | 2.5 |
|  | 2 Yes, every day and of sufficient quantity | 7.5 |
| **2. Is a reliable safe drinking water station present and accessible for staff, patients and families at all times and in all locations/wards?**  CIRCLE ONE ANSWER | 0 No, not available | 0 |
|  | 1 Sometimes, or only in some places or not available for all users | 2.5 |
|  | 2 Yes, accessible at all times and for all wards/groups | 7.5 |
| **Hand hygiene and sanitation facilities** | | |
| **3. Are functioning hand hygiene stations (that is, alcohol-based hand rub solution or soap and water and clean single-use towels) available at all points of care?**  CIRCLE ONE ANSWER | 0 No, not present | 0 |
|  | 1 Yes, stations present, but supplies are not reliably available | 2.5 |
|  | 2 Yes, with reliably available supplies | 7.5 |
| **4. In your facility, are ≥ 4 toilets or improved latrines28 available for outpatient settings or ≥ 1 per 20 users for inpatient settings?**  CIRCLE ONE ANSWER | 0 Less than required number of toilets or latrines available and functioning | 0 |
|  | 1 Sufficient number present but not all functioning | 2.5 |
|  | 2 Sufficient number present and functioning | 7.5 |
| **Power supply, ventilation and cleaning** | | |
| **5. In your health care facility, is sufcient energy/power supply available at day and night for all uses (for example, pumping and boiling water, sterilization and decontamination, incineration or alternative treatment technologies, electronic medical devices, general lighting of areas where health care procedures are performed to ensure safe provision of health care and lighting of toilet facilities and showers)? CIRCLE ONE ANSWER** | 0 No | 0 |
|  | 1 Yes, sometimes or only in some of the mentioned areas | 2.5 |
|  | 2 Yes, always and in all mentioned areas | 5 |
| **6. Is functioning environmental ventilation (natural or mechanical29) available in patient care areas?** | 0 No | 0 |
|  | 1 Yes | 5 |
| **7. For ﬂoors and horizontal work surfaces, is there an accessible record of cleaning, signed by the cleaners each day?**  CIRCLE ONE ANSWER | 0 No record of floors and surfaces being cleaned | 0 |
|  | 1 Record exists, but is not completed and signed daily or is outdated | 2.5 |
|  | 2 Yes, record completed and signed daily | 5 |
| **8. Are appropriate and well-maintained materials for cleaning (for example, detergent, mops, buckets, etc.) available?**  CIRCLE ONE ANSWER | 0 No materials available | 0 |
|  | 1 Yes, available but not well maintained | 2.5 |
|  | 2 Yes, available and well-maintained | 5 |
| **Patient placement and personal protective equipment (PPE) in health care settings** | | |
| **9. Do you have single patient rooms or rooms for cohorting30 patients with similar pathogens if the number of isolation rooms is insufficient (for example, TB, measles, cholera, Ebola, SARS)?**  CIRCLE ONE ANSWER | 0 No | 0 |
|  | 1 No single rooms but rather rooms suitable for patient cohorting available | 2.5 |
|  | 2 Yes, single rooms are available | 7.5 |
| **10. Is PPE32 available at all times and in sufcient quantity for all uses for all health care workers?**  CIRCLE ONE ANSWER | 0 No | 0 |
|  | 1 Yes, but not continuously available in sufficient quantities | 2.5 |
|  | 2 Yes, continuously available in sufficient quantities | 7.5 |
| **Medical waste management and sewage** | | |
| **11. Do you have functional waste collection containers for non-infectious (general) waste, infectious waste and, sharps waste in close proximity to all waste generation points?**  CIRCLE ONE ANSWER | 0 No bins or separate sharps disposal | 0 |
|  | 1 Separate bins present but lids missing or more than 3/4 full; only two bins (instead of three); or bins at some but not all waste generation points | 2.5 |
|  | 2 Yes | 5 |
| **12. Is a functional burial pit/fenced waste dump or municipal pick-up available for disposal of non-infectious (non-hazardous/ general waste)?**  CIRCLE ONE ANSWER | 0 No pit or other disposal method used | 0 |
|  | 1 Pit in facility but insufficient dimensions; pits/dumps overfilled or not fenced/locked; or irregular municipal waste pick up | 2.5 |
|  | 2 Yes | 5 |
| **13. Is an incinerator or alternative treatment technology for the treatment of infectious and sharp waste (for example, an autoclave) present (either present on or off site and operated by a licensed waste management service), functional and of a sufficient capacity?**  CIRCLE ONE ANSWER | 0 No, none present | 0 |
|  | 1 Present, but not functional | 1 |
|  | 2 Yes | 5 |
| **14. Is a wastewater treatment system (for example, septic tank followed by drainage pit) present (either on or off site) and functioning reliably?** | 0 No, not present | 0 |
|  | 1 Yes, but not functioning reliably | 2.5 |
|  | 2 Yes and functioning reliably | 5 |
| **Decontamination and sterilization** | | |
| **15. Does your health care facility provide a dedicated decontamination area and/or sterile supply department (either present on or off site and operated by a licensed decontamination management service) for the decontamination and sterilization of medical devices and other items/equipment?**  CIRCLE ONE ANSWER | 0 No, not present | 0 |
|  | 1 Yes, but not functioning reliably | 2.5 |
|  | 2 Yes and functioning reliably | 5 |
| **16. Do you reliably have sterile and disinfected equipment ready for use?**  CIRCLE ONE ANSWER | 0 No, available on average < five days per week | 0 |
|  | 1 Yes, available on average ≥ five days per week or every day, but not of sufficient quantity | 2.5 |
|  | 2 Yes, available every day and of sufficient quantity | 5 |
| **17. Are disposable items available when necessary? (for example, injection safety devices, examination gloves)**  CIRCLE ONE ANSWER | 0 No, not available | 0 |
|  | 1 Yes, but only sometimes available | 2.5 |
|  | 2 Yes, continuously available | 5 |
| **Subtotal score** **/100** | | |

| **Interpretation: A three-step process** | |
| --- | --- |
| 1. Add up your points | |
|  | Score |
| Section (Core component) | Subtotals |
| 1. IPC programme |  |
| 1. IPC guidelines |  |
| 1. IPC education and training |  |
| 1. HAI surveillance |  |
| 1. Multimodal strategies |  |
| 1. Monitoring/audits of IPC practices and feedback |  |
| 1. Workload, staffing and bed occupancy |  |
| 1. Built environment, materials and equipment for IPC at the facility level |  |
| **Final total score /800** | |
|  | |
